# Associations of dietary macronutrients and energy intake with diabetic kidney disease and all-cause mortality in diabetes: evidence from NHANES

**DOI:** 10.1136/bmjdrc-2026-005941

**Published:** 2026-09-30

**Authors:** Qiuhong Li, Xiaomeng Lv, Liuwei Wang, Yulin Wang, Yanhong Guo, Lu Yu, Zihan Zhai, Zhibin Huang, Lin Tang

**Affiliations:** 1 Department of Nephrology, The First Affiliated Hospital of Zhengzhou University, Zhengzhou, China; 2 The First Affiliated Hospital of Zhengzhou University, Zhengzhou, China

**Keywords:** Energy Intake, Diet, Diabetic, Kidney Diseases

## Abstract

**Introduction:**

Dietary management is crucial for diabetes, yet the impact of macronutrient and energy intake on diabetic kidney disease (DKD) and all-cause mortality remains unclear. This study aimed to investigate these associations and to identify intake levels that were associated with the lowest odds of DKD and mortality.

**Research design and methods:**

Using combined cross-sectional and prospective data from NHANES (2011–2018), we analyzed 3066 diabetic adults (representing 30.3 million individuals in the USA). Weighted logistic regression was used to assess the prevalence of DKD, while Cox regression was employed to evaluate associations with all-cause mortality, incorporating dose-response analyses and mediation testing.

**Results:**

After multivariable adjustment, higher protein and dietary fiber intake showed significant inverse linear associations with DKD (P-trend <0.05). Compared with the lowest quartile (Q1), the highest quartile (Q4) had significantly lower DKD odds: protein (OR=0.41, 95% CI 0.21 to 0.79) and fiber (OR=0.50, 95% CI 0.31 to 0.82). The statistical inflection points associated with lower DKD odds were protein >74 g/day and fiber >14 g/day. Exploratory mediation analysis suggested that body mass index and hemoglobin partially explained the associations of proteins and fibers with DKD, respectively. Prospectively, after 48 months median follow-up (366 deaths, representing 3.36 million), only energy intake exhibited a significant U-shaped association with mortality risk (non-linear p<0.05). Mortality was lowest with daily energy intake between 1750 kcal and 1810 kcal.

**Conclusions:**

In this observational analysis, higher protein (>74 g/day) and fiber intake (>14 g/day) were associated with lower DKD prevalence in diabetes, while maintaining daily energy within 1750–1810 kcal is associated with the lowest all-cause mortality risk. These findings suggest potential reference values for dietary patterns in diabetes management.

**WHAT IS ALREADY KNOWN ON THIS TOPIC**

- Dietary management is fundamental in diabetes, but the intake levels of macronutrients and energy that are associated with the lowest diabetic kidney disease (DKD) odds and mortality are unclear.

**WHAT THIS STUDY ADDS**

- In this nationally representative US diabetic population, protein intake >74 g/day and fiber >14 g/day were associated with lower DKD odds, while a U-shaped association was found for energy intake, with the lowest all-cause mortality risk at 1750–1810 kcal/day. Body mass index and hemoglobin partially mediated the relationship of proteins and fibers with DKD.

**HOW THIS STUDY MIGHT AFFECT RESEARCH, PRACTICE, OR POLICY**

- These findings provide exploratory reference values for dietary patterns in diabetes management, suggesting that moderate increases in protein and fiber, alongside maintaining energy balance, could be associated with lower DKD prevalence and all-cause mortality risk in diabetes. Future randomized trials are needed to confirm causality.

## Background

 Diabetes affects 537 million adults globally, with predictions indicating a rise to 783 million by 2045.^1^ Diabetic kidney disease (DKD), affecting 20%–40% of patients with diabetes, is the leading cause of new dialysis patients.^2^ The all-cause mortality risk for people with diabetes is 1.89 times higher than that for people without diabetes,^3^ imposing severe healthcare burdens. Dietary management is an important component of basic diabetes treatment.^4^ Dietary management is fundamental for diabetes care, as macronutrients (protein, carbohydrates, fiber, fat, sugar) and energy intake critically influence DKD development and mortality through metabolic and inflammatory pathways.^5^

Although the importance of energy and macronutrients in diabetes management has been widely recognized, most studies have focused on the general population, and results remain controversial. Protein effects are debated due to divergent impacts of animal/plant sources^5^ and unclear mortality links.^6–8^ Carbohydrates may accelerate DKD via hyperfiltration and glycation,^9 10^ yet their mortality association is inconsistent.^11–13^ Dietary fiber shows renal and anti-inflammatory benefits.^14 15^ Studies have also shown that a high intake of total dietary fiber is associated with reduced inflammation and mortality risks.^16 17^ Fat intake correlates with higher mortality.^18^ Energy imbalance drives DKD pathogenesis. While sugar exacerbates renal damage,^19 20^ research indicates a connection between sugary beverage intake and all-cause mortality, but it lacks an association with chronic kidney disease (CKD) risk.^21 22^ Energy imbalance drives DKD pathogenesis,^23 24^ yet the intake levels associated with the lowest DKD prevalence and mortality among patients with diabetes remain unclear.

Additionally, particularly regarding a single dietary component such as carbohydrates,^25–28^ protein,^29 30^ or fat.^31–33^ No studies have simultaneously examined the association of these nutrients and energy intake with the incidence of DKD and mortality in patients with diabetes. Our study aims to fully leverage the large sample size and long-term follow-up data of the NHANES database to systematically investigate the association of these nutrients and energy intake with DKD prevalence and all-cause mortality in patients with diabetes, as well as to identify intake levels that were associated with the lowest risk in this observational study and elucidate mediating mechanisms. This will provide exploratory evidence for informing more precise dietary intervention plans for patients with diabetes and potentially contribute to improved long-term prognosis and quality of life.

## Methods

### Study populations

Between 2011 and 2018, NHANES data were downloaded, which is a nationally representative cross-sectional study conducted every 2 years by the National Center for Health Statistics (NCHS) to assess the health and nutrition status of adults and children. Approximately 5000 random samples are selected annually using a multistage, stratified probability sampling design. The participants were evaluated nutritionally and physically at Mobile Examination Centers (MECs) using standardized interviews, physical examinations, and laboratory tests. There is detailed free statistics available at https://www.cdc.gov/nchs/nhanes/.^34^

Among 39 156 participants in the NHANES 2011–2018, we excluded 35 266 participants without diabetes, 28 under the age of 18 years, 796 with missing energy, protein, carbohydrate, total sugars, dietary fiber, total fat, and mortality status data. Eventually, a total of 3066 participants were enrolled in the study. The detailed procedure for selecting participants was shown in figure 1.

**Figure 1:**
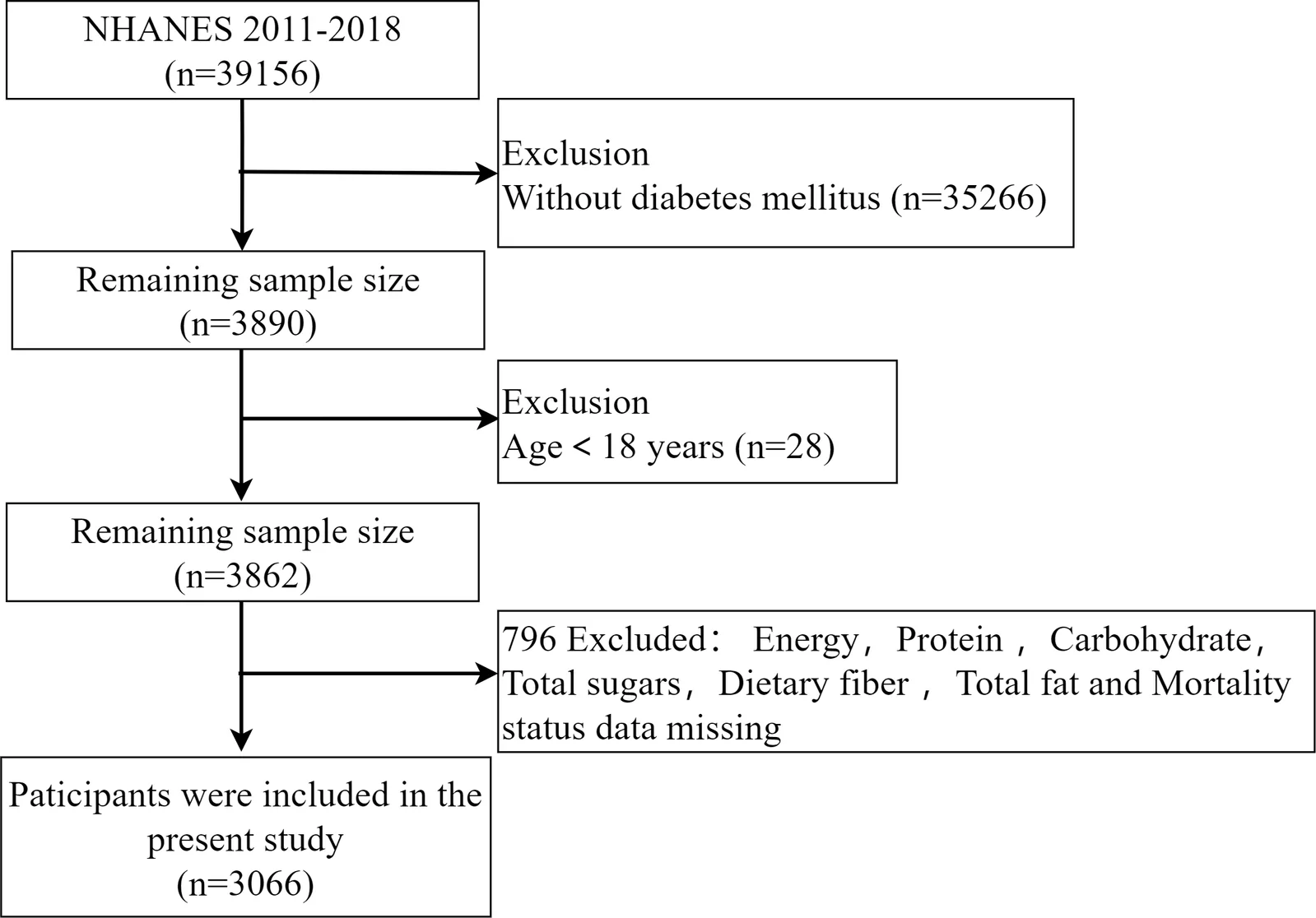
Flow chart of subject selection for this study.

### Covariates

In this study, covariates that may affect the relationship of protein, carbohydrate, total sugars, dietary fiber, total fat, and energy with DKD and mortality were included. Demographic covariates including gender, age, race; health risk factors including smoking status, drinking status, hypertension, cardiovascular disease (CVD), congestive heart failure (CHF); physical examination including body mass index (BMI); laboratory tests including white blood cell (WBC), hemoglobin (HGB), blood platelet (PLT), urinary albumin-creatinine ratio (ACR), blood urea nitrogen (BUN), serum creatinine (SCR), serum uric acid (SUA), glucose (GLU), glycated hemoglobin (HbA1c), aspartate aminotransferase (AST), alanine aminotransferase (ALT), plasma albumin (ALB), total cholesterol (TC), low-density lipoprotein cholesterol (LDL-C), plasma triglyceride, and ratio of family income to poverty (INDFMPIR).

### Dietary macronutrients and energy

All NHANES participants are eligible for two 24-hour dietary recall interviews. The first dietary recall interview is collected in-person in the MEC, and the second interview is collected by telephone 3–10 days later. The dietary intake data are used to estimate the types and amounts of foods and beverages (including all types of water) consumed during the 24-hour period prior to the interview (midnight to midnight), and to estimate intakes of energy, nutrients, and other food components from those foods and beverages. In this study, the average dietary data from two 24-hour dietary recall interviews were used to calculate the intakes of protein, carbohydrate, total sugars, dietary fiber, total fat, and energy.

### Definition of covariates

In the study, diabetes was defined as (1) self-reported previous diagnosis of diabetes by a physician, (2) fasting plasma glucose ≥7.0 mmol/L, (3) HbA1c ≥6.5 mmol/L, or (4) taking diabetes medications.

### Main outcome

DKD was defined using cross-sectional NHANES data as estimated glomerular filtration rate (eGFR, calculated using the Chronic Kidney Disease Epidemiology Collaboration formula) <60 mL/min/1.73 m^2^ and/or ACR (≥30 mg/g) in patient with diabetes. Because NHANES provides only single-time-point measurements, this definition reflects the presence of renal abnormalities at a single time point and does not confirm chronicity as required in clinical diagnostic criteria.

Another outcome variable was mortality status, which was determined by using the National Death Index (NDI) by 31 December 2019. The NDI is a highly reliable and widely used resource for death identification. The international classification of Diseases, 10th Revision was used to determine disease-specific death. There are also free detailed statistics available at https://www.cdc.gov/nchs/linked-data/mortality.

### Statistical analysis

To account for the complex NHANES survey design, appropriate weights, stratification, and primary sampling units were applied to ensure nationally representative estimates. Continuous variables were presented as mean±SD if they were approximately normally distributed, and median and IQR if they were skewed. Categorical variables were presented as a number (%). The weighted variance (ANOVA) or Kruskal-Wallis test (continuous variables) and weighted χ² test (categorical variables) were used to compare differences in baseline characteristics divided by different groups by survival status. The relationships of macronutrients and energy with DKD were analyzed using a sampling-weighted multivariate logistic regression model (results presented as ORs and 95% CI). The associations of macronutrients and energy with the risk of all-cause mortality events were analyzed using a sampling-weighted multivariate Cox proportional risk regression (results are expressed as HRs and 95% CI). The dose-response relationships of macronutrients and energy with DKD and mortality were analyzed using restricted cubic spline (RCS) curves. Furthermore, exploratory mediation analysis (1000 bootstrap resamples) was performed to estimate the proportion of protein/BMI and fiber/HGB associations with DKD that could be explained by these potential mediators. However, given the cross-sectional design of the DKD analysis, temporal ordering cannot be established. The results are therefore exploratory and hypothesis-generating and should not be interpreted as evidence of causal mediation.

All the statistical analyses were performed by R software V.4.3.2. Two-tailed p values <0.05 were considered statistically significant.

## Results

### Baseline characteristics

A total of 3066 diabetes cases representing 30 262 233.42 US in diabetes were included in our study. Among them, patients with DKD account for approximately 37.61%. At a median follow-up of 48 months, a total of 366, representing 3 363 466.63 (12.5%) all-cause mortalities, were recorded. Characteristics of the sample with weighted participant numbers stratified by the survival status are shown in table 1. At baseline, the mean age was 59.56±9.97 years, 51.84% of participants (representing 15.69 million US adults) were men, and 48.16% of participants (representing 14.57 million US adults) were women. Compared with the surviving participants, those who had passed away were more likely to be older and have higher levels of ACR, BUN, SCR, and SUA. They were also more likely to have a history of CVD, CHF, DKD, while concurrently demonstrating lower levels of HGB, PLT, ALT, ALB, LDL-C, INDFMPIR, energy, protein, and total fat. Additionally, they were less likely to have a history of drinking and hypertension. The differences were significant (p<0.05).

**Table 1:** Baseline characteristics of participants according to mortality status

Characteristic	All-cause mortality		P value
	No	Yes	
n	26 898 766.79	3 363 466.63	
Age (years)	59.00 (50.00–68.00)	72.00 (62.00–80.00)	<0.001
Male, n (%)	13 870 156.0 (51.6)	1 819 157.5 (54.1)	0.573
Race (%)			
Mexican American	2 845 359.8 (10.6)	224 266.7 (6.7)	0.015
Other Hispanic	1 870 547.4 (7.0)	134 307.5 (4.0)	
Non-Hispanic white	15 683 318.6 (58.3)	2 332 970.5 (69.4)	
Non-Hispanic black	3 815 518.3 (14.2)	467 867.2 (13.9)	
Non-Hispanic Asian	1 699 846.7 (6.3)	67 072.6 (2.0)	
Other race	984 176.1 (3.7)	136 982.1 (4.1)	
Smoking, n (%)	3 791 987.5 (29.8)	419 800.8 (19.9)	0.081
Drinking, n (%)	20 208 197.9 (76.9)	2 287 502.9 (68.8)	0.033
Hypertension, n (%)	9 388 782.9 (34.9)	692 329.9 (20.7)	0.002
CVD, n (%)	2 722 230.1 (10.2)	763 140.0 (23.0)	<0.001
CHF, n (%)	1 791 532.3 (6.7)	741 981.2 (22.3)	<0.001
BMI (kg/m^2^)	32.30 (28.40–37.30)	31.45 (26.60–37.60)	0.218
WBC (*10^9^/L)	7.60 (6.40–9.10)	7.80 (6.50–9.00)	0.302
HGB (g/dL)	14.20 (13.10–15.10)	13.50 (12.10–14.44)	<0.001
PLT (*10^9^/L)	228.00 (190.00–277.00)	210.00 (166.78–259.00)	0.001
ACR (mg/g)	11.23 (6.47–27.66)	39.36 (11.75–158.01)	<0.001
BUN (mmol/L)	5.36 (3.93–6.78)	6.72 (4.64–9.28)	<0.001
SCR (µmol/L)	76.02 (62.76–91.94)	102.10 (75.14–122.88)	<0.001
SUA (µmol/L)	327.10 (273.60–392.60)	380.70 (305.87–426.71)	<0.001
GLU (mmol/L)	7.05 (5.72–9.44)	7.38 (6.15–10.02)	0.214
HbA1c (%)	6.70 (6.20–7.80)	6.70 (6.20–7.80)	0.885
ALT (U/L)	23.00 (17.00–32.00)	20.00 (14.00–24.00)	<0.001
AST (U/L)	22.00 (18.00–29.00)	23.00 (19.00–31.00)	0.155
ALB (g/L)	42.00 (39.00–44.00)	40.00 (38.00–42.00)	<0.001
TC (mmol/L)	4.63 (3.93–5.40)	4.42 (3.69–5.32)	0.214
LDL-C (mmol/L)	2.61 (2.02–3.28)	2.30 (1.76–2.97)	0.019
TG (mmol/L)	1.87 (1.25–2.68)	1.90 (1.29–2.71)	0.9
INDFMPIR	2.65 (1.27–4.67)	1.77 (1.03–3.29)	<0.001
Energy (kcal)	1852.79 (1417.00–2374.52)	1780.11 (1230.24–2253.36)	0.012
Protein (g)	74.30 (55.85–97.90)	68.82 (50.81–88.48)	0.02
Carbohydrate (g)	215.66 (159.03–273.57)	200.91 (142.22–284.02)	0.256
Total sugars (g)	80.63 (53.61–117.08)	82.82 (53.95–136.17)	0.421
Dietary fiber (g)	15.40 (11.25–21.20)	14.64 (10.30–19.25)	0.112
Total fat (g)	74.71 (53.07–100.49)	68.16 (41.77–91.56)	0.005
DKD (%)	8 772 561.1 (32.6)	2 608 531.0 (77.6)	

Data are presented as the mean±SE or median (IQR) for continuous variables, and the numbers (%) for categorical variables.

P<0.05 was considered statistically significant.

ACR, urinary albumin-creatinine ratio; ALB, plasma albumin; ALT, alanine aminotransferase; AST, aspartate aminotransferase; BMI, body mass index; BUN, blood urea nitrogen; CHF, congestive heart failure; CVD, cardiovascular disease; DKD, diabetic kidney disease; GLU, glucose; HbA1c, glycated hemoglobin; HGB, hemoglobin; INDFMPIR, ratio of family income to poverty; LDL-C, low-density lipoprotein cholesterol; PLT, blood platelet; SCR, serum creatinine; SUA, serum uric acid; TC, total cholesterol; TG, plasma triglyceride; WBC, white blood cell.

### Relationship of dietary macronutrients and energy intake with DKD

The results of sampling-weighted multivariate logistic regression analyses to reveal the associations between protein, carbohydrate, total sugars, dietary fiber, total fat, energy, and DKD are shown in table 2. Although there was an association in unadjusted models, after adjusting for age, gender, INDFMPIR, HGB, HbA1c, ALT, AST, BMI, TC, smoking, drinking, CHF, CVD, hypertension history at baseline, neither energy nor total fat levels were found to be associated with DKD. However, in the unadjusted variable model, there were lower DKD odds in Q2, Q3, Q4 of protein (0.78 (0.60 to 1.01), 0.87 (0.61 to 1.23), 0.49 (0.34 to 0.70)), dietary fiber (0.92 (0.70 to 1.22), 0.81 (0.57 to 1.14), 0.62 (0.46 to 0.85)), compared with Q1 (p for trend <0.05). After adjusting for covariates, there were still lower DKD odds in Q2, Q3, Q4 of protein (0.71 (0.36 to 1.41), 0.73 (0.37 to 1.42), 0.41 (0.21 to 0.79)), dietary fiber (0.80 (0.47 to 1.37), 0.76 (0.42 to 1.37), 0.50 (0.31 to 0.82)), compared with Q1 (p for trend <0.05).

**Table 2:** Multivariate logistic regression analysis on the relationship of dietary macronutrients and energy intake with DKD

	Model 1		Model 2		Model 3	
	OR (95% CI)	P value	OR (95% CI)	P value	OR (95% CI)	P value
Quartiles of protein		0.001		0.036		0.032
Quartile 1	Reference		Reference		Reference	
Quartile 2	0.78 (0.60 to 1.01)		0.80 (0.61 to 1.05)		0.71 (0.36 to 1.41)	
Quartile 3	0.87 (0.61 to 1.23)		0.92 (0.64 to 1.32)		0.73 (0.37 to 1.42)	
Quartile 4	0.49 (0.34 to 0.70)		0.55 (0.37 to 0.84)		0.41 (0.21 to 0.79)	
Quartiles of carbohydrate		0.100		0.200		0.5
Quartile 1	Reference		Reference		Reference	
Quartile 2	0.89 (0.61 to 1.30)		0.90 (0.62 to 1.32)		1.11 (0.63 to 1.96)	
Quartile 3	0.81 (0.57 to 1.14)		0.82 (0.58 to 1.15)		0.91 (0.54 to 1.53)	
Quartile 4	0.62 (0.41 to 0.95)		0.68 (0.45 to 1.04)		0.68 (0.34 to 1.35)	
Quartiles of total sugars		0.2		0.10		0.4
Quartile 1	Reference		Reference		Reference	
Quartile 2	0.70 (0.49 to 0.99)		0.63 (0.44 to 0.91)		0.61 (0.32 to 1.17)	
Quartile 3	0.81 (0.57 to 1.13)		0.74 (0.50 to 1.09)		0.84 (0.47 to 1.49)	
Quartile 4	0.76 (0.52 to 1.11)		0.77 (0.51 to 1.15)		0.76 (0.37 to 1.56)	
Quartiles of dietary fiber		0.019		0.016		0.047
Quartile 1	Reference		Reference		Reference	
Quartile 2	0.92 (0.70 to 1.22)		0.86 (0.64 to 1.15)		0.80 (0.47 to 1.37)	
Quartile 3	0.81 (0.57 to 1.14)		0.74 (0.52 to 1.06)		0.76 (0.42 to 1.37)	
Quartile 4	0.62 (0.46 to 0.85)		0.59 (0.42 to 0.83)		0.50 (0.31 to 0.82)	
Quartiles of total fat		0.035		0.2		0.088
Quartile 1	Reference		Reference		Reference	
Quartile 2	0.77 (0.54 to 1.08)		0.78 (0.54 to 1.12)		0.89 (0.49 to 1.62)	
Quartile 3	0.77 (0.53 to 1.11)		0.77 (0.52 to 1.15)		0.74 (0.37 to 1.45)	
Quartile 4	0.61 (0.43 to 0.86)		0.67 (0.46 to 0.97)		0.52 (0.31 to 0.90)	
Quartiles of energy		0.033		0.2		0.055
Quartile 1	Reference		Reference		Reference	
Quartile 2	0.85 (0.60 to 1.19)		0.87 (0.62 to 1.24)		1.46 (0.91 to 2.34)	
Quartile 3	0.79 (0.57 to 1.09)		0.85 (0.60 to 1.20)		1.07 (0.63 to 1.80)	
Quartile 4	0.58 (0.40 to 0.84)		0.65 (0.44 to 0.97)		0.66 (0.40 to 1.09)	

Model 1: no adjustment.

Model 2: adjusted for age, gender.

Model 3: adjusted for all the factors in model 2 and INDFMPIR, HGB, HbA1c, ALT, AST, BMI, TC, smoking, drinking, CHF, CVD, and hypertension at baseline.

ALT, alanine aminotransferase; AST, aspartate aminotransferase; BMI, body mass index; CHF, congestive heart failure; CVD, cardiovascular disease; DKD, diabetic kidney disease; HbA1c, glycated hemoglobin; HGB, hemoglobin; INDFMPIR, ratio of family income to poverty; TC, total cholesterol.

### Relationship of dietary macronutrients and energy intake with all-cause mortality

At a median follow-up of 48 months, a total of 366, representing 3 363 466.63 (12.5%) deaths, were recorded. Table 3 presents the association between protein, carbohydrate, total sugars, dietary fiber, total fat, energy, and the risk of all-cause mortality. Although elevated protein, total fat, and energy levels were all associated with lower mortality risk in the unadjusted model (all p<0.05). After adjusting for confounding factors (age, gender, INDFMPIR, HGB, SCR, ACR, UA, ALB, race, HbA1c, ALT, AST, BMI, TC, CHF, CVD, hypertension at baseline), only energy was independently associated with the all-cause mortality event, with HRs (95% CIs) in Q2, Q3, Q4 of 0.49 (0.31 to 0.80), 0.92 (0.52 to 1.64), and 0.74 (0.41 to 1.36), compared with Q1 (p<0.05).

**Table 3:** Multivariate Cox regression analysis on the relationship of dietary macronutrients and energy intake with all-cause mortality

	Model 1		Model 2		Model 3	
	HR (95% CI)	P value	HR (95% CI)	P value	HR (95% CI)	P value
Quartiles of protein		0.006		0.3		0.7
Quartile 1	Reference		Reference		Reference	
Quartile 2	0.70 (0.49 to 1.01)		0.77 (0.55 to 1.08)		0.78 (0.48 to 1.25)	
Quartile 3	0.72 (0.46 to 1.12)		0.79 (0.50 to 1.27)		1.07 (0.64 to 1.79)	
Quartile 4	0.48 (0.31 to 0.74)		0.72 (0.48 to 1.08)		0.89 (0.58 to 1.37)	
Quartiles of carbohydrate		0.079		0.07		0.5
Quartile 1	Reference		Reference		Reference	
Quartile 2	0.86 (0.61 to 1.23)		0.91 (0.63 to 1.32)		0.95 (0.57 to 1.57)	
Quartile 3	0.62 (0.44 to 0.89)		0.70 (0.49 to 0.99)		0.82 (0.49 to 1.35)	
Quartile 4	0.79 (0.53 to 1.17)		1.12 (0.74 to 1.71)		1.18 (0.73 to 1.91)	
Quartiles of total sugars		0.2		0.007		0.13
Quartile 1	Reference		Reference		Reference	
Quartile 2	0.94 (0.62 to 1.42)		0.82 (0.56 to 1.21)		0.95 (0.53 to 1.69)	
Quartile 3	0.76 (0.48 to 1.22)		0.72 (0.45 to 1.14)		0.91 (0.50 to 1.66)	
Quartile 4	1.22 (0.77 to 1.96)		1.39 (0.85 to 2.26)		1.48 (0.80 to 2.73)	
Quartiles of dietary fiber		0.2		0.2		0.50
Quartile 1	Reference		Reference		Reference	
Quartile 2	0.77 (0.53 to 1.11)		0.72 (0.50 to 1.04)		0.72 (0.47 to 1.10)	
Quartile 3	0.87 (0.54 to 1.40)		0.84 (0.53 to 1.34)		0.96 (0.53 to 1.75)	
Quartile 4	0.60 (0.37 to 0.97)		0.67 (0.43 to 1.05)		0.92 (0.58 to 1.44)	
Quartiles of total fat		0.001		0.039		0.055
Quartile 1	Reference		Reference		Reference	
Quartile 2	0.56 (0.37 to 0.86)		0.61 (0.41 to 0.91)		0.51 (0.30 to 0.84)	
Quartile 3	0.71 (0.46 to 1.09)		0.84 (0.55 to 1.30)		0.84 (0.48 to 1.47)	
Quartile 4	0.47 (0.30 to 0.74)		0.68 (0.43 to 1.07)		0.72 (0.42 to 1.24)	
Quartiles of energy		<0.001		0.008		0.029
Quartile 1	Reference		Reference		Reference	
Quartile 2	0.49 (0.32 to 0.75)		0.53 (0.37 to 0.78)		0.49 (0.31 to 0.80)	
Quartile 3	0.71 (0.43 to 1.18)		0.86 (0.50 to 1.46)		0.92 (0.52 to 1.64)	
Quartile 4	0.43 (0.27 to 0.70)		0.65 (0.40 to 1.04)		0.74 (0.41 to 1.36)	

Model 1: no adjustment.

Model 2: adjusted for age, gender.

Model 3: adjusted for all the factors in model 2 and INDFMPIR, HGB, SCR, ACR, UA, ALB, race, HbA1c, ALT, AST, BMI, TC, CHF, CVD, hypertension at baseline.

ACR, urinary albumin-creatinine ratio; ALB, plasma albumin; ALT, alanine aminotransferase; AST, aspartate aminotransferase; BMI, body mass index; CHF, congestive heart failure; CVD, cardiovascular disease; HbA1c, glycated hemoglobin; HGB, hemoglobin; INDFMPIR, ratio of family income to poverty; SCR, serum creatinine; TC, total cholesterol; UA, uric acid.

### Dose-response relationship of dietary macronutrients and energy intake with DKD and all-cause mortality

The RCS curve analyses revealed that protein and dietary fiber were linearly negatively correlated with DKD (non-linear p>0.05) after controlling for confounders, and the cut-off points were about 74 g and 14 g, respectively (figure 2a, b, respectively).

**Figure 2:**
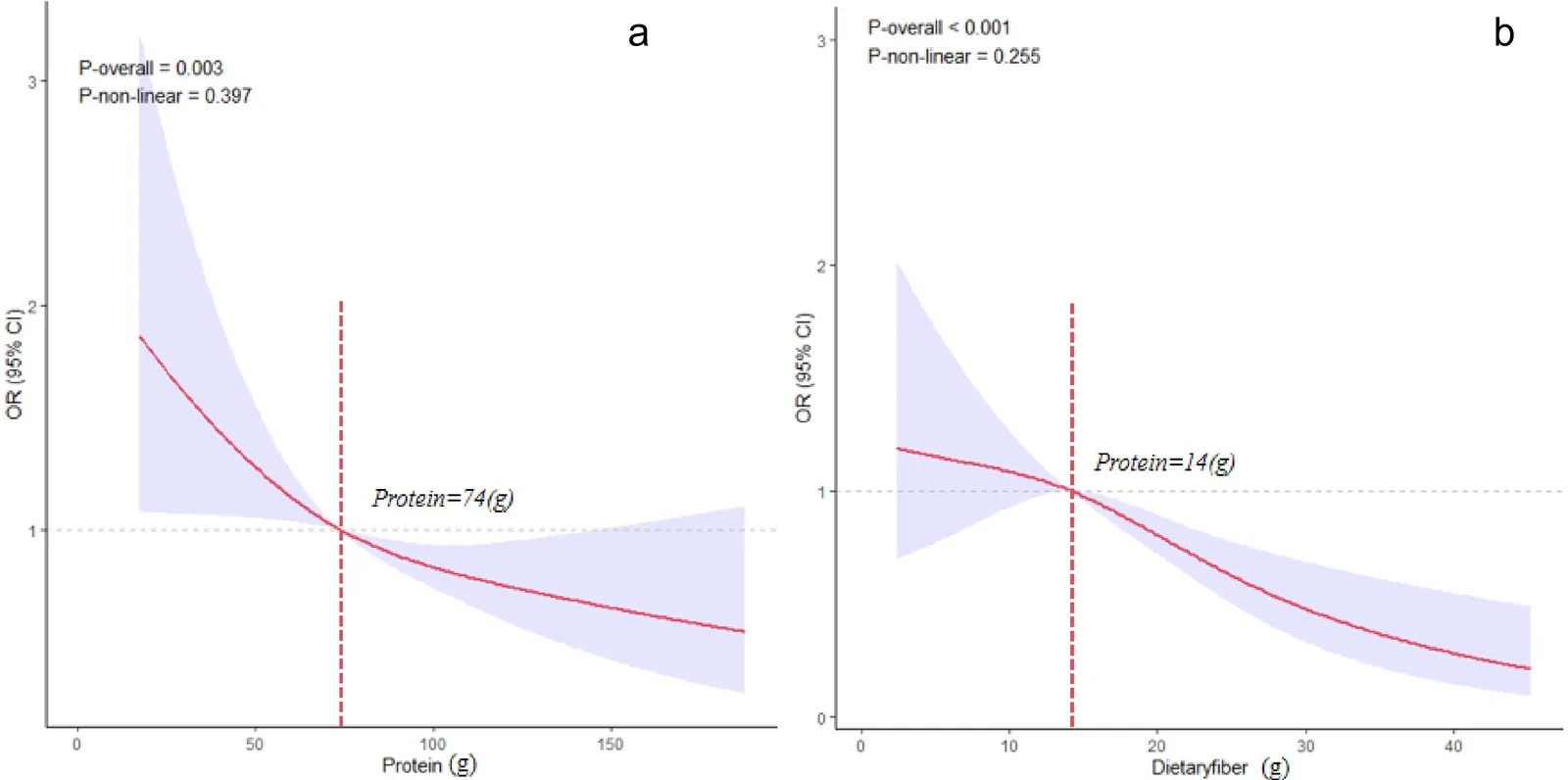
(a) Restricted cubic spline curves analyses of protein and DKD. (b) Restricted cubic spline curves analyses of dietary fiber and DKD. All adjusted for age, gender, INDFMPIR, HGB, HbA1c, ALT, AST, BMI, TC, smoking, drinking, CHF, CVD, hypertension at baseline. ALT, alanine aminotransferase; AST, aspartate aminotransferase; BMI, body mass index; CHF, congestive heart failure; CVD, cardiovascular disease; DKD, diabetic kidney disease; HbA1c, glycated hemoglobin; HGB, hemoglobin; INDFMPIR, Ratio of family income to poverty; TC, total cholesterol.

However, energy level showed a U-shaped relationship with the risk of all-cause mortality (non-linear p<0.05). The statistical inflection points were about 1750 kcal and 1810 kcal (figure 3).

**Figure 3:**
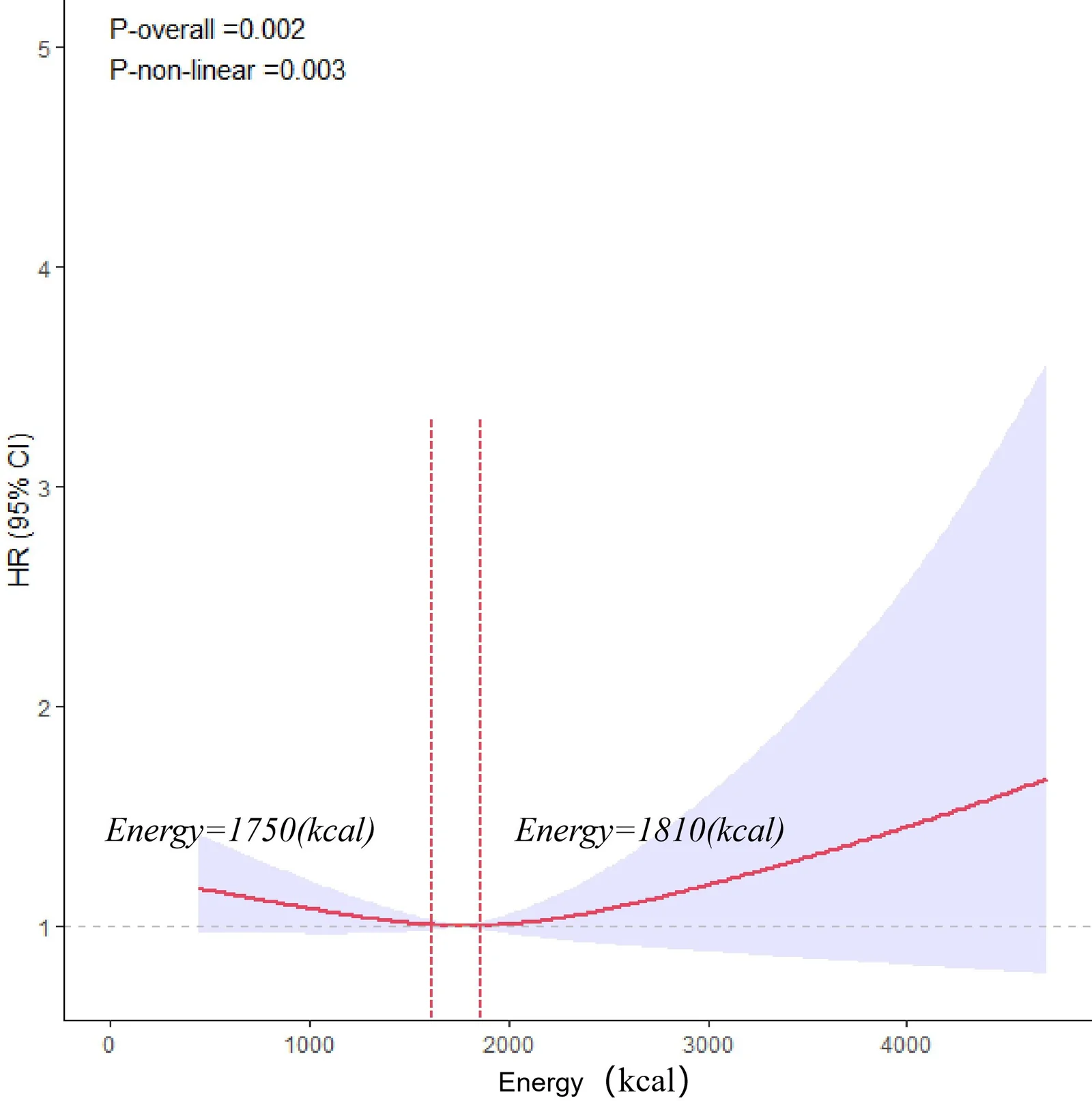
Restricted cubic spline curves analyses of energy and all-cause mortality. All adjusted for age, gender, INDFMPIR, HGB, SCR, ACR, UA, ALB, race, HbA1c, ALT, AST, BMI, TC, CHF, CVD, hypertension at baseline. ACR, urinary albumin-creatinine ratio; ALB, plasma albumin; ALT, alanine aminotransferase; AST, aspartate aminotransferase; BMI, body mass index; CHF, congestive heart failure; CVD, cardiovascular disease; HbA1c, glycated hemoglobin; HGB, hemoglobin; INDFMPIR, ratio of family income to poverty; SCR, serum creatinine; TC, total cholesterol; UA, uric acid.

### The mediation effects in the relationships of dietary macronutrients and energy intake with DKD and all-cause mortality

Mediation analyses were conducted to explore mediators that mediate the association of protein and dietary fiber exposure with DKD (figure 4a, b, respectively). BMI statistically accounted for approximately 9.5% of the association between protein intake and DKD, and HGB accounted for approximately 8.0% of the association between dietary fiber intake and DKD. Regrettably, we have not identified any intermediate variables in the relationship between energy and all-cause mortality.

**Figure 4:**
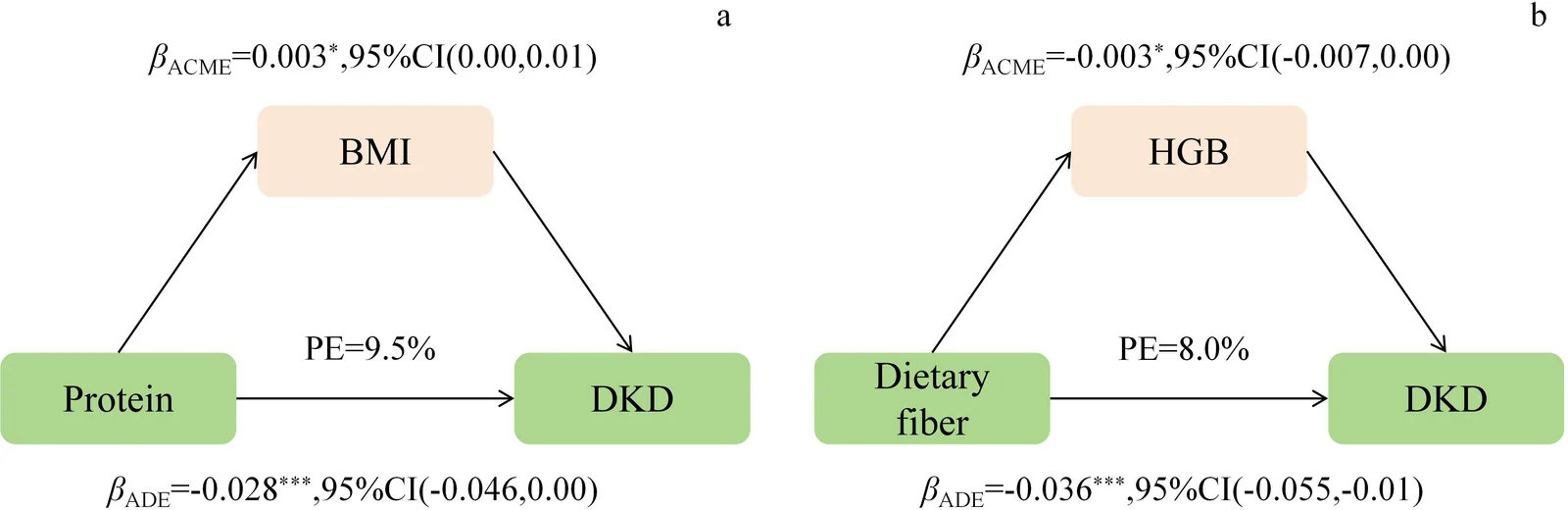
(a) The mediation effects in the relationships of protein with DKD. (b) The mediation effects in the relationships of dietary fiber with DKD. The model was adjusted for age, gender, INDFMPIR, HGB, HbA1c, ALT, AST, BMI, TC, smoking, drinking, CHF, CVD, hypertension at baseline. ALT, alanine aminotransferase; AST, aspartate aminotransferase; BMI, body mass index; CHF, congestive heart failure; CVD, cardiovascular disease; DKD, diabetic kidney disease; HbA1c, glycated hemoglobin; HGB, hemoglobin; INDFMPIR, ratio of family income to poverty; TC, total cholesterol.

## Discussion

To our knowledge, this is the first cross-sectional and prospective cohort study to investigate the associations of dietary macronutrients and energy intake with DKD and all-cause mortality in individuals with diabetes. In this cohort of adults aged 18 years and older individuals, our analyses revealed that higher levels of protein and dietary fiber intake were associated with lower odds of DKD. Specifically, daily protein intake above 74 g and fiber intake above 14 g were associated with lower DKD odds. Furthermore, we identified that BMI and HGB could partially elucidate these associations. Additionally, energy intake exhibited a U-shaped association with mortality risk, with the lowest mortality observed among those consuming between 1750 and 1810 kcal/day. These findings offer exploratory evidence for identifying dietary patterns that are most strongly associated with DKD and all-cause mortality among individuals with diabetes, thereby highlighting the potential importance of dietary interventions in managing these high-risk populations.

This study found that high protein intake was associated with lower DKD odds in patients with diabetes, but not with an increased mortality risk, which is consistent with previous studies.^7^ There have also been studies that have found a high protein intake is associated with a lower risk of death,^35 36^ while other studies have found a positive correlation between total protein intake and all-cause mortality.^37^ The different results may be that our study focused on patients with diabetes, while some studies included a broader range of patients with CKD and attributed more benefits to plant protein intake. Our study found that a daily protein intake of more than 74 g was associated with lower DKD odds, while previous studies have shown that protein intake should account for 15% to 20% of total energy intake for patients with normal kidney function.^38^ Based on an energy intake of 1800 kcal/day, this translates to roughly 68–90 g/day, aligning with our research findings. It is worth noting that our exploratory mediation analysis revealed that BMI accounted for small proportions of the associations of protein intake with DKD, suggesting that protein may be related to improved body composition, thereby maintaining muscle mass.^39^ Muscle tissue is a major site for GLU disposal and can improve insulin sensitivity. Second, moderate protein consumption may regulate appetite hormones, such as the anorexigenic hormones peptide YY (PYY) and glucagon-like peptide-1 (GLP-1), leading to increased satiety and aiding in weight management and metabolic control.^40^ Another possible explanation is that a high protein intake produces more bioactive peptides. These peptides are known to exhibit anti-inflammatory, anti-hypertensive, anti-oxidative, and anti-microbial effects,^41^ leading to lower DKD odds. However, because the DKD analysis is cross-sectional, we cannot establish temporal ordering between dietary intake, BMI, and DKD. Therefore, these mediation findings should be considered hypothesis-generating rather than evidence of causal pathways.

Our research findings on the protective association of dietary fiber with the kidneys align with existing studies.^42–44^ For example, in three large cohorts, those with the highest fiber intake had a 40%–50% lower prevalence of CKD.^44–46^ Additionally, the Tehran Lipid and Glucose Study (n=1630) found an 11% reduced risk of developing CKD for every 5 g/day increase in total fiber intake.^43^ While the relationship between fiber intake and risk of death varies among different populations.^43 45 47 48^ Following fiber-rich, plant-based diets is linked to lower all-cause mortality risk in the general population.^47 48^ Evidence suggests this link is stronger in individuals with non-dialysis CKD than those without CKD.^16 43 49^ However, some studies have not found a connection between fiber intake and mortality risk in patients with hemodialysis^50^ or peritoneal dialysis^51^. Our study found no relationship between fiber intake and mortality in patients with diabetes. This may be because we did not distinguish sources of fiber. The fiber threshold associated with lower DKD odds identified in our study (14 g/day) is lower than most current recommendations.^52–54^ Most countries now recommend adults consume 25–35 g of fiber daily, based on robust trials and large cohort studies on fiber supplements and fiber-rich diets.^55^ Although there are differences, it is worth noting that NHANES data shows that the average American adult consumes fiber of only 15–18 g/day,^56^ while the average Chinese resident consumes around 11 g/day. The vast majority of people do not reach the recommended standard.

The protective mechanisms of dietary fiber may be more varied. Chronic inflammation is a key driver of the progression of DKD and can lead to functional iron deficiency and anemia. Dietary fiber enhances excretion and regulates the intestinal microbiota, reducing the production and absorption of toxins such as indoxyl sulfate, and increasing the production of short-chain fatty acids (SCFAs). This, in turn, reduces systemic inflammation, improves intestinal barrier function, and regulates GLU and lipid metabolism, thereby promoting kidney health.^57^ Moreover, fiber slows down gastric emptying and GLU absorption, enhancing postprandial glycemic control and diminishing the harm caused by GLU toxicity to the kidneys.^58^ Fiber can also bind and promote the excretion of bile acids, thereby improving lipid metabolism and providing indirect protection to the kidneys. Most importantly, our exploratory mediation analysis highlighted that HGB statistically accounted for a portion of the association between fiber intake and DKD, suggesting a potential link between fiber intake, anemia status, and kidney function. Possible pathways include the role of dietary fiber in intestinal health and iron utilization, the effect of SCFAs on systemic inflammation and erythropoietin production, and the relationship between glycemic control and HbA1c levels. These potential mechanisms may help explain the observed association between fiber intake, anemia, and a lower odds of DKD.

The U-shaped relationship between energy intake and mortality is consistent with several previous studies.^59–61^ However, our study also differs from some others. A study targeting the elderly population indicated that higher total energy intake was associated with a lower risk of all-cause mortality.^62^ Another study involving elderly individuals from Spain indicated that higher energy intake was linked to an increased risk of mortality, especially from cardiovascular causes.^63^ Some studies have also shown that there is no significant association between total energy intake and all-cause mortality.^64 65^ These discrepancies in study outcomes may stem from differences in study populations and sample sizes. Our research underscores the significance of energy balance in the management of diabetes. Excessive energy intake leads to obesity, insulin resistance, chronic inflammation, and oxidative stress,^66^ which accelerate the progression of diabetes complications; conversely, insufficient energy intake may be associated with loss of lean body mass, malnutrition, and impaired immune function.^67^ Notably, the energy range (1750–1810 kcal) associated with the lowest mortality is close to the recommended intake for the Chinese diet (2000 kcal)^68^ and US diet (1000–3200 kcal).^69^ The recommended daily energy intake is not a fixed value because it needs to be adjusted dynamically based on factors such as age, gender, activity level, and disease status. In addition, lower energy intake may reflect underlying frailty or illness rather than a causal protective effect; residual reverse causation cannot be completely ruled out in the energy-mortality association. However, these threshold values are statistical inflection points derived from a spline model and should not be interpreted as precise clinical targets due to dietary measurement limitations.

Our study possesses several strengths. First, it uses high-quality dietary data from a representative population-based study (NHANES) to investigate the relationship of macronutrient and energy intake with DKD as well as all-cause mortality in patients with diabetes. Second, it uses various statistical methods to comprehensively evaluate the correlation between macronutrients and health outcomes in patients with diabetes. It establishes a dose-response relationship between nutrients and DKD and reveals the mediating effects of BMI and HGB, thereby providing insights into potential mechanisms. Third, the associations reported in this study have been adjusted for various important confounding factors, ensuring the stability and reliability of the observed relationships.

However, it is important to acknowledge certain limitations. First, the study is based on an observational, retrospective analysis of existing databases, which restricts causal inference. Second, although total macronutrient intake should be taken into account, we analyzed absolute intakes without energy adjustment in the primary analysis. We will apply energy-adjusted models in our further exploratory studies. Third, multiple comparisons increase type I error risk. While trend and dose-response analyses support our main findings, chance findings cannot be ruled out. Replication in studies with pre-specified hypotheses and correction for multiple testing is needed. Fourth, dietary intake was assessed using only two 24-hour recalls, which are subject to within-person variability and recall bias. This may cause non-differential misclassification and regression dilution bias.

## Conclusion

In this observational study based on NHANES data, higher protein intake (>74 g/day) and fiber intake (>14 g/day) were associated with lower DKD odds in patients with diabetes. Additionally, daily energy intake within approximately 1750–1810 kcal was associated with the lowest all-cause mortality risk. These findings provide suggestive evidence that balanced macronutrient and energy intake may be relevant to DKD and mortality in patients with diabetes. Further research, including randomized controlled trials, is needed to clarify whether these associations reflect causal effects and to develop individualized nutritional intervention strategies.

## Data Availability

Data are available in a public, open access repository.
